# Real-World Evidence: The Low Validity of Temperature Screening for COVID-19 Triage

**DOI:** 10.3389/fpubh.2021.672698

**Published:** 2021-06-30

**Authors:** Bogdan C. Pană, Henrique Lopes, Florentina Furtunescu, Diogo Franco, Anca Rapcea, Mihai Stanca, Alina Tănase, Anca Coliţă

**Affiliations:** ^1^Department of Public Health, University of Medicine and Pharmacy Carol Davila Bucharest, Bucharest, Romania; ^2^Public Health Unit, Institute of Health Sciences, Universidade Católica Portuguesa (Catholic University of Portugal), Lisbon, Portugal; ^3^Bone Marrow Transplantation Unit, Fundeni Clinical Institute, Bucharest, Romania; ^4^Department of Pediatrics, University of Medicine and Pharmacy Carol Davila Bucharest, Bucharest, Romania

**Keywords:** triage, screening, non-contact temperature measurement, COVID-19, SARS-CoV-2

## Abstract

**Background:** The COVID-19 pandemic forced health-related organizations to rapidly launch country-wide procedures that were easy to use and inexpensive. Body temperature measurement with non-contact infrared thermometers (NCITs) is among the most common procedures, both in hospital settings and in many other entities. However, practical hospital experiences have raised great doubts about the procedure's validity.

**Aim:** This study aimed to evaluate the validity of the body temperature measured using NCITs among oncological and transplant patients who took the polymerase chain reaction test for SARS-Cov-2 PCR+ and PCR- in a Romanian Hospital.

**Methods:** Body temperature was measured for 5,231 inpatients using NCITs. The cutoff point for fever was equal to or above 37.3°C. Patients then completed a questionnaire about their symptoms, contact, and travel history.

**Findings:** Fever was detected in five of 53 persons with PCR+, resulting in a sensitivity of 9.43% (95% CI, 3.13–20.66%). No fever was verified in 5,131 of 5,171 persons with PCR-, resulting in a specificity of 99.15% (95% CI, 98.86–99.38%). A defensive vision of NCIT procedure (maximum standard error only in favor) had a sensitivity of 15.09% (95% CI, 6.75–27.59%).

**Conclusions:** The use of NCITs in a triage provides little value for detection of COVID-19. Moreover, it provides a false sense of protection against the disease while possibly discriminating individuals that could present fever due to other reasons, such as oncologic treatments, where fever is a common therapeutical consequence. The consumption of qualified human resources should be considered, especially in the context of the shortage of healthcare professionals worldwide.

## Introduction

The infectious disease COVID-19, caused by the SARS-CoV-2 virus, has been widely disseminated globally, with over 40 million infection cases and over 1.1 million deaths registered during the pandemic (1). In Romania, 324,094 cases and 8,389 deaths due to COVID-19 have been reported since the beginning of the pandemic, that is, from February 26 to November 11, 2020 (1). During April–August 2020, the monthly average of new cases varied from 334 in April to 230 in May and 248 in June. Romania declared a state of emergency (lockdown) on March 16, which ended on May 14 but was followed by a state of alert. The monthly average of new cases reached 477 in July and 721 in August 2020.

Although the most common symptoms of the disease (fever, dry cough, and tiredness, among others) are mild (2), it has been reported that asymptomatic people may be transmission vectors for the disease. Asymptomatic rates have a broad variability ranging from 5 to 80% (3).

In Romania, each hospital carries out procedures for screening, testing, and patient management (Appendix A in Supplementary Material) based on periodical recommendations issued by the National Center for Surveillance and Control of Transmittable Disease (NCSCTB) of the National Institute of Public Health from Romania (NIPH) (4, 5).

These procedures change according to the pandemic's evolution, access to new findings and evidence on symptoms (including measurement of temperature), travel history, contact history, and criteria for different categories of patients or medical personal to qualify for PCR testing. Recommendations by the Center for Disease Control and Prevention (CDC) and European Centre for Disease Prevention and Control (ECDC) (6, 7) are implemented for symptom assessment for COVID-19 in new and returning patients along with practitioners and visitors, including the daily measurement of body temperature for all patients in healthcare settings.

Globally, massive non-contact temperature screening is used in hospitals, malls, office buildings, airports, and so on, as a fundamental test in the COVID-19 triage for clinical, epidemiologic, and other public health reasons. According to the U.S. Food & Drug Administration (FDA) (8), the normal body temperature should range between 36.1 to 37.2°C with the use of non-contact infrared thermometers (NCITs). However, the actual behavior of this procedure remains to be proved in the field. It is imperative to know the extent to which it is an effective and safe method, or whether it leads to false clues or bias against patients—particularly in oncology and transplant patients where fever may occur for multiple reasons. It must be noted that various factors may influence measurement of body temperature while screening for fever (e.g., environmental conditions, antipyretics, and other respiratory diseases) (9). For example, there are treatments in oncology and immunocompromised states that may lead to fever. In these cases, a positive measurement in patients who visit oncology/transplant hospitals may be due to a priori fever associated with their condition, leading to a potentially erroneous measurement of body temperature. A study in a radiation oncology center in China, from January to April, identified 27 cases of fever in a total of 770 patients with cancer (10). A case study of four COVID-19 patients with cancer in China reported that all of them registered fever 6–36 days after hospital admission (11). Another study, with a cohort of 138 COVID-19 inpatients in a Chinese hospital, regardless of cancer status, reported that 20% of these patients had a fever below 38°C (12).

One study reported that fever was registered in ~43% of COVID patients at the time of admission to hospital and in ~89% by the time of hospitalization (13). The median duration of fever in these patients was between 8 and 11 days (14).

In many cancer patients with fever, the symptom could be due to febrile neutropenia (FN) or even a flu-like syndrome (15).

Cancer patients are at a higher risk of any infection due to immunosuppression, commonly caused by cancer and cancer-related treatment. Thus, they are at a higher risk of COVID-19 infection than the general population. Patients receiving active cancer treatment are at a higher risk than those in remission.

Fever is also a common clinical manifestation in patients who have had a transplant, as well as for other clinical reasons not related to COVID-19. Although the purpose of hematopoietic stem cell transplant (HSCT) is curative, and the patient could suffer damage if the procedure is delayed, treatment-related toxicities and technical difficulties would be amplified and overwhelming during this pandemic. Therefore, professional societies such as the European Society for Blood and Marrow Transplantation (EBMT) have issued guidelines to help physicians during the pandemic. These include sorting and testing patients (including those without symptoms) before being admitted to a transplant ward and allocating separate areas, distinct from the transplant units, for symptomatic patients awaiting COVID-19 test results. There are also strict rules for stem cell donors.

Highly immunocompromised pediatric and adult hematopoietic cell transplant (HCT) recipients frequently experience respiratory infections caused by viruses that are less virulent in immunocompetent individuals (16).

Respiratory viral infections in allogeneic HCT recipients contribute to significant mortality rates ranging between 10 and 50% if the infection progresses to the lower respiratory tract (17).

Another dimension that should be discussed is that fever is a non-specific, non-sensitive indicator of infection. In developing countries, infections are the major cause of a fever of unknown origin (18, 19).

In Romania, each hospital/medical center allocates resources for screening with questionnaires and body temperature. The Clinical Institute Fundeni (CIF) is one of the few hospitals in the country doing PCR tests for all inpatients, in view of its specific oncology and transplants profile, and is aligned with similar hospitals in Europe.

We aim to provide evidence for the low validity of the body temperature measurement method as it has the possibility of inducing error and stigmatizing people without COVID-19. This will allow people infected with COVID-19 to pass through the triage with a false sense of safety.

The primary objective of this study was to evaluate the sensitivity of fever measurement with NCITs for positive SARS-Cov-2 test results that identified the disease in the first PCR+ testing. The secondary objective was to determine the specificity and accuracy of the fever measurement with NCITs.

## Methods

### Setting

The study was conducted at the Clinical Institute Fundeni, a major referral hospital in Bucharest, the capital city of Romania, with core competencies on transplant (medullar, liver, renal) and oncology. The Clinical Institute Fundeni has had an average of 53,060 inpatients in the last 5 years, with more than 80% being oncological and transplant patients. The study was conducted in accordance with the Declaration of Helsinki, the protocol was approved by the Ethics Committee of Clinical Institute Fundeni, and the patients provided informed consent.

### Sample

The sample consisted of 5,231 patients with age ranging from 6 months to 91 years (M = 53.97, SD = 17.95). The sample included both female (47%) and male (53%) individuals, and was highly representative (assuming equal probability of fever over the year), with CI of 95 ± 1.30% and CI of 99 ± 1.71%.

**Figure 1:**
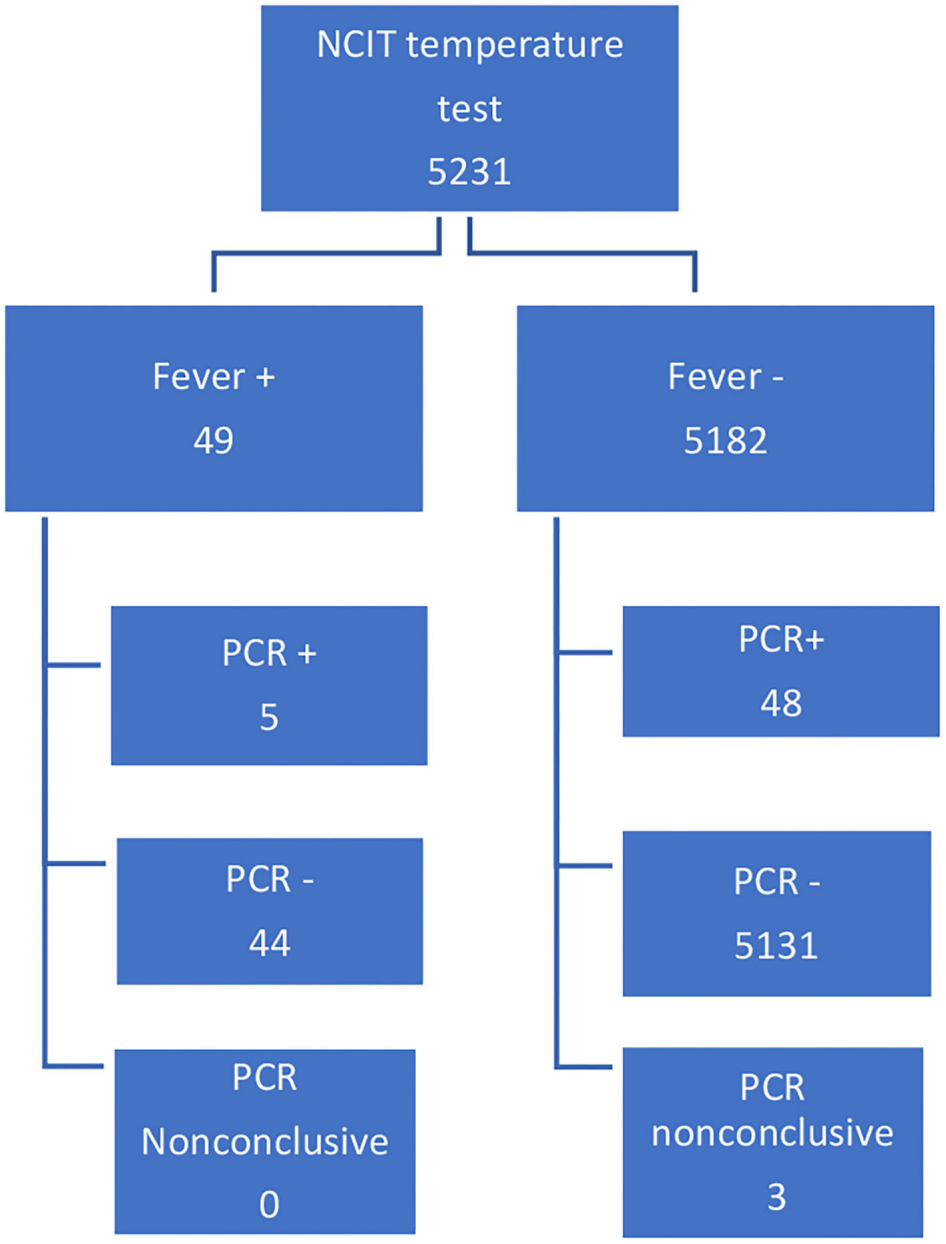
Patients selection.

All patients tested for SARS-CoV-2 from March 16 to August 30, 2020 were eligible for inclusion. Patients with non-conclusive PCR in the initial testing were not included (N-3 cases); see Figure 1. All patients with at least 1 day of hospitalization were considered. Consultations, visits, or accompanying patients were excluded from the sample.

### Design

This was a consecutive observational study encompassing all patients admitted to the hospital from March to August 2020.

### Procedure

Temperature was measured on the day of admission in the triage tents placed in front of the hospital entrances with NCITs held at a distance of one to five centimeters from the skin.

Along with the temperature measurements, a questionnaire about symptoms, contacts, and travel history was completed by patients. Data were collected for body temperature at the time of testing, age, sex, admission department, criteria for testing measurement, symptoms, contacts, and travel history (Supplementary Material 1).

In line with the FDA recommendation (8), fever was defined as a ≥37.3°C body temperature.

The triage data recording system included a handwritten paper registry completed by the triage teams, trained and designated by the hospital.

After the triage, all admitted patients were allocated to one of three zones according to the triage's findings for nose and throat swabs—green (no findings for COVID-19), yellow (suspicions for COVID-19), or red (possibly positive for COVID-19).

Testing of nose and throat swab samples was performed using a quantitative reverse-transcription polymerase chain reaction in the CIF laboratory.

Nasal and oropharyngeal samples from patients to be hospitalized at the Fundeni Clinical Institute were collected within a single tube of universal transport medium (UTM^®^ Copan Italia S.p.A., Brescia, Italy) to prevent viral RNA degradation and bacterial/fungal overgrowth. Briefly, 200 μl of the sample was processed with a Seegene Nimbus automated system, which performs both RNA extraction–using STARMag 96 × 4 Viral DNA/RNA 200 C Kit—and PCR assay setup. The RNA isolation procedure comprised four steps: sample lysis, binding nucleic acid to magnetic beads, washing debris, and purified nucleic acid elution. The Allplex 2019-nCoV Assay (Seegene, Seoul, South Korea) is an *in vitro* diagnostic (IVD) real-time reverse transcriptase-polymerase chain reaction (RT-PCR) assay developed for the detection of the nucleic acids in human samples from individuals with symptoms of severe acute respiratory syndrome related to coronavirus 2 (SARS-CoV-2). This assay was designed for amplifying three viral targets: the E gene (specific to the subgenus Sarbecovirus), as well as the N and the RdRP genes (both specific to SARS-CoV-2) (20). The tube PCR strips with the extracted RNA was loaded onto a real-time PCR C1000 CFX96 Thermal Cycler (Bio-Rad Laboratories, Hercules, CA, USA). Positive and negative controls were included in each run. After completion of the assay, the Seegene Viewer 2019-nCoV software allowed automated analysis and interpretation of results.

After receiving the PCR results, the patient was finally allocated to the department's red or green zone. Data from the laboratory were available in an Excel format. Two resident doctors worked for 2 months to manually match registered temperatures with PCR results in the Microsoft Excel software.

### Quantitative Analysis Tools

Results were reported using proportions with 95% confidence intervals. All analyses were conducted with SPSS version 25. Calibration is not necessary for the devices mentioned, eliminating the need to include the calculation or the calibration error or to verify the calibration date and accreditation of the calibrating entity. The devices used are authorized for operation without calibration, and certified for medical or private use for measuring human or animal temperature by the FDA (Certificate of conformity number 3015697152), European Metrology (CE) (Certificate of compliance number 4M200326.SZTDD37), and ISO13485: 2016 (Certificate of compliance number TW50598U, issued on April 8, 2019).

## Results

Of the 5,231 individuals, 49 tested positive for fever (fever +), and 53 were PCR +. Three PCR tests were inconclusive, and the remaining 5,175 were negative. For fever or PCR, there was no significant difference between males and females. Out of the 53 PCR + cases, five registered a fever, but 48 did not. Out of the 5,175 PCR—cases, 44 registered a fever (Figure 1). Sensitivity 9.43%, 95% CI (3.13–20.66%). Specificity 99.15% 95% CI (98.86–99.38%). Accuracy 98.25%, 95% CI (97.86–98.59%). Fisher exact <0.001. Disease prevalence 1.00%.

## Discussion

A sensitivity value of 9.5% at the first testing of PCR+ is considered to be extremely low. This means that a significant number of SARS-CoV-2 patients would pass through the temperature triage and be admitted to the hospital as *negative* when, in fact, they would be *positive*—suggesting the possibility of a large number of *false negatives*. Consequently, the effort and cost of mobilizing staff to measure temperatures at the entrance increase all nosocomial risks since more than 90% of people with COVID-19 end up proceeding with the clinical process. The value of this NCIT method for the daily assessment of COVID-19 inpatients was not evaluated.

Our findings suggest that 48 patients, representing 90.5% of PCR + patients (1—sensitivity), were *false negatives*. Patients were admitted to a green, yellow, or red zone of the department, following triage findings based on symptoms, history, contact, and temperature. The internal code for this procedure was PO-MED-40. The PCR was performed within 6–24 h, depending on the status of the medical emergency. The hospital also conducted the PCR on all admitted patients, even those who did not fulfill the criteria (289 of 5,321).

Specificity value was found to be very high, reaching 99.15%. Nonetheless, 44 patients out of 5,228 were *false positive* (1—specificity). This implies delay in medical services for these patients, at least until the PCR tests were conducted.

It must be noted that no calculation was done for the whole hospital triage scheme in place, which also included questions about contact, symptoms, and travel history. The results may vary if these questions are added to the temperature triage. The cut-off point of 37.3°C considered for fever was another concern.

Our findings are consistent with the previous literature (21, 22). A study in an Australian hospital concluded that fever screening lacked sensitivity to detect patients with SARS-CoV-2 (23). In another study set in a hospital, out of 40,887 patients who attended health services, fever was detected in five patients on the outdoor triage and in 37 patients in the indoor clinic zone after being acclimatized (24). Therefore, screening for fever in Taiwan hospitals needs to be reinforced, with body temperature measured in two separate time slots and zones (24), allowing for acclimatization to each environment, which could otherwise mask the presence of fever. This was considered an important step to reduce the risk of admitting individuals with a fever that was not detected at the first screening.

Although body temperature measurement provides some help in identifying patients with COVID-19, its use involves some risk, as follows:

- The vast majority of PCR + cases are not identified. Besides providing a false sense of security, people who are not infected are set in the same space as those who are infected, possibly for hours.
- Most people who are referred to the red zone are false positives, generating stigmatization and great anxiety in most cases, which are harmful for patients who are already fragile due to their condition.
- We suggest studying screening alternatives other than NCITs in the context of COVID-19. Once the resistance to rapid testing based on the most recent bibliography (about the generation of false negatives) is overcome, use of rapid PCR is a possibility. Although its safety level is lower than that of serologic tests, it is much more sensitive than screening based on body temperature measurement.

For future research directions, it would be interesting to use the same assessment to verify whether fevers, false positives, and false negatives occur in other types of hospital services with the same density as they do in cancer and transplant hospitals. We estimate the probability of fever results directly from concrete phases of treatment, as well as with the type of cancer. However, it would be important to verify and study patients who should always be excluded from this type of procedure. Recognizing that there is no reliable method, it would be interesting to compare the validity of fever measurement, with or without insertion in a broader protocol, by using rapid PCR tests in another sample. Finally, we believe it is of the utmost importance to complement this work with an economic study involving the direct and indirect costs of this procedure. Costs include labor, use of space and equipment, and, in particular, risk of contagion outbreaks in the hospital from unidentified COVID-19 cases.

## Conclusion

Our findings indicate that testing for fever with NCITs has a very low sensitivity to COVID-19, thus questioning NCIT use for fever screening. The issue is not about the utility of the device (for metrological reading) but its use in clinical testing procedures (patient safety reading).

Not only is the usefulness of NCIT-based testing open to question, but it might induce a false sense of security while reducing the patient's effective safety in hospital settings as it allows passage of COVID-19 patients. It also diverts attention from public health measures that are more likely to be effective uses of resources, such as self-isolation when ill, physical distancing, mask-wearing, and contact tracing.

the context of the severe shortage of qualified personnel to respond to needs of the growing number of cases in the pandemic while catering to the demands of the current healthcare setting, the fever-screening procedure increases financial costs and personnel allocation needs with no apparent gain.

Therefore, we recommend that body temperature measurement be eliminated from hospital admission procedures, especially in oncological hospitals or other hospitals with high immunological impairment.

A possible alternative solution for screening patients for admission to hospital could be rapid low-cost testing for a first triage while serological PCR results are determined. It also allows for a quick response to the circulation of people within the hospital with a greater degree of safety than currently verified.

## Data Availability Statement

The datasets generated for this study can be found in online repositories. The names of the repository/repositories and accession number(s) can be found below: https://osf.io/grsqh/?view_only=dc293a57a99a4d21bb0fd138e75cc7d7/.

## Ethics Statement

The studies involving human participants were reviewed and approved by Ethics Council of the Fundeni Clinical Institute. Written informed consent to participate in this study was provided by the participants' legal guardian/next of kin.

## Author Contributions

BP, FF, AT, and AC contributed to conceptualization of the study. BP, HL, FF, AT, and AC contributed toward the methodology. Software was organized by BP and validation was done by BP, HL, and FF. BP and HL performed the formal analysis, and investigation was carried out by HL, DF, AR, MS, AT, and AC. AT and AC gathered the resources, while BP, AR, and MS curated the data. The original draft was prepared by BP, HL, FF, DF, AT, and AC. HL, FF, DF, AT, and AC reviewed and edited the manuscript, while HL supervised. BP administered the project. All authors have read and agreed to the published version of the manuscript.

## Conflict of Interest

The authors declare that the research was conducted in the absence of any commercial or financial relationships that could be construed as a potential conflict of interest.

## Abbreviations

CDC: Centers for Disease Control and Prevention
CIF: Clinical Institute Fundeni
EBMT: The European Society for Blood and Marrow Transplantation
ECDC: European Centre for Disease Prevention and Control
FDA: U.S. Food & Drug Administration
FN: Febrile Neutropenia
HCT: Hematopoietic Cell Transplant
HSCT: Hematopoietic Stem Cell Transplant
CI: Confidence Interval
IVD: *In Vitro* Diagnostic
NCIT: Non-contact Infrared Thermometers
NCSCTB: National Center for Surveillance and Control of Transmittable Disease
NIPH: National Institute of Public Health
PCR: Polymerase Chain Reaction
RNA: Ribonucleic Acid
UTM: Universal Transport Medium.
