# Supplementary material for: Real-World Evidence: The Low Validity of Temperature Screening for COVID-19 Triage
Source: Front Public Health. 2021 Jun 30;9:672698. doi: 10.3389/fpubh.2021.672698 (PMC8277959; doi:10.3389/fpubh.2021.672698)
Supplement: Supplementary file 1 [file Table_1.DOCX]

Supplementary Material

# Appendix A

Fundeni Clinical Institute

Questionnaire template for COVID-19 disease risk assessment of CIF patients.

Number…………...

Register number………...

Date …. /……/2020

Name/Surname……………

PRN……………… ……………….

Temperature at entrance……………… (Celsius degrees)

1. Please answer the following questions with YES or NO.

- Are you a person living/or lived with a COVID-19 patient in the last 14 days? **YES/NO**
- Did you have physical contact with a COVID-19 patient in the last 14 days (hand shaking unfollowed by hand hygiene)? **YES/NO**
- Did you have unprotective contact with COVID-19 patient secretions (for example, touching a handkerchief without glows)? **YES/NO**
- Did you have face-to-face contact with a COVID-19 infected person at a distance shorter than 2 meters and for more than 15 minutes in the last 14 days? **YES/NO**
- Have you been in the same meeting room/waiting room with a COVID-19 patient for more than 15 minutes and at a distance shorter than 2 meters in the last 14 days?  **YES/NO**
- Are you medical personnel caring for COVID-19 patients, or a laboratory person handling COVID-19 patients probes, without wearing protective equipment in the last 14 days? **YES/NO**

1. Did you have a sudden onset of one or some of the following symptoms: **cough, fever, shortness of breath** (increasing of the respiration frequency) **YES/NO**
2. Please confirm if you are in one of the following situations that implicates home isolation*:

- You traveled in the last 14 days in places with COVID-19, other than those with extensive community transmission? **YES/NO**
- You had direct contact with confirmed COVID-19 positive persons? **YES/NO**
- Your family members are in one of the above situations? **YES/NO**

Completed by…………

Signature……………………...

*Accordingly, with article 1, paragraph (3) and (4) from Order nr.414/2020 regarding quarantine for persons under international COVID -19 situations, persons under point 3 incidence have to isolate themselves at home for 14 days and to monitor health status.
